# The effect of mental and physical health problems on sickness absence

**DOI:** 10.1007/s10198-021-01379-w

**Published:** 2021-10-09

**Authors:** Mark L. Bryan, Andrew M. Bryce, Jennifer Roberts

**Affiliations:** Department of Economics, University of Sheffield, Sheffield, UK

**Keywords:** Absenteeism, Mental health, Physical health, Labour market, Labour force survey, C23, I10, J01

## Abstract

Absenteeism is an important feature of the labour market, imposing significant costs on employers and the economy as a whole. This paper is the first to use a large labour force survey sample to investigate how different physical and mental health conditions affect absence rates among prime age workers in the UK. A pooled time series/cross-section analysis reveals that people with a chronic health condition are more likely to be absent from work, and mental health has a significantly larger effect than physical health. From a longitudinal perspective, we find that a change in mental health has an effect on absenteeism more than three times greater than a change in physical health. These findings imply that the prevention and alleviation of chronic health conditions, particularly common mental disorders such as depression and anxiety that are highly prevalent in prime age workers, will deliver significant benefits to the UK economy due to reduced absenteeism. Further, there is significant heterogeneity between different health conditions, with some having no effect at all on absenteeism having controlled for other factors.

## Introduction

Absenteeism is an important feature of the labour market. It is estimated that employees in the UK took an average of 4.1 sickness days in 2017; although this has declined substantially since records began in 1993. As we might expect, workers with chronic health problems have substantially higher rates of absence than others: the percentage of working hours lost due to sickness absences in 2017 was 3.9% for people with a chronic health condition, compared to 1.2% for other workers [1].

The financial burden of absenteeism falls largely on employers, with one estimate suggesting that the median cost of absence per employee was £522 in 2016 [2]. However, individuals will also bear some cost if absence results in reduced income. Sickness absence may be necessary to accelerate recovery and prevent further health deterioration and, as highlighted in the COVID-19 pandemic, prevent the spread of disease to the wider workforce. However, absenteeism also denies individuals the wider benefits of work, such as improved health and well-being [3].

Despite the importance of sickness absence as a feature of the labour market, we know very little about its relationship with underlying health. This study is the first to estimate the extent to which different chronic health conditions affect sickness absence in the UK. We go beyond the raw statistics reported by the Office for National Statistics (ONS) [1], which may be confounded by other different personal and work characteristics, and provide evidence to show how the onset of specific physical and mental health conditions impact on absenteeism.

We find that people with a chronic health condition are more likely to be absent from work, with mental health having a significantly larger effect than physical health. From a longitudinal perspective, a change in mental health has an effect on absenteeism more than three times greater than a change in physical health. These findings imply that the prevention and alleviation of chronic health conditions, particularly common mental disorders such as depression and anxiety that are highly prevalent in prime age workers, will deliver significant benefits to the UK economy due to reduced absenteeism. Further, there is significant heterogeneity between different health conditions, with some having no effect at all on absenteeism, once other factors are controlled for.

It may seem obvious that health should be the primary determinant of sickness absence. However, individuals experience a wide range of different health conditions and each one presents unique risks to absenteeism. As shown in Table 1, a person’s long-term health condition is not necessarily the same or related to their reason for absence, suggesting that health or disability is not itself a direct determinant of absence. For example, the most common reason for absence among those with a mental health problem is stress, depression or anxiety but this reason accounts for less than a third of absence in this group. Further, health is not the only determinant of sickness absence; other individual and work characteristics must also be taken into account. Some recent international research found that absence rates in Australia are about 5% higher among workers with poor mental health compared to those with good mental health, and that this is mitigated somewhat by increased job control and, for women, increased job security [4]. It has also been found that having a long-term health condition leads to a significantly higher rate of sickness absence in Australia, and this effect is higher for men and workers experiencing low job control [5]. In a study of six European countries (excluding the UK), people with severe disabilities had higher absence than non-disabled people, even controlling for indicators of short-term health state. However, those with moderate disabilities had lower absence than non-disabled people [6]. Danish evidence also suggests that transitions between work and sickness absence are greater for those with chronic illness [7]. Evidence from Sweden, however, shows that increasing expenditure on healthcare does not lead to reduced sickness absence [8].

**Table 1:** Reason for absence by type of chronic health condition

Reason for sickness absence	All absences (%)	Chronic health condition	
		Any physical health condition (%)	Any mental health condition (%)
Back pain	6.5	9.6	5.4
Neck and upper limb problems	4.0	5.3	3.2
Other musculoskeletal problems	7.5	10.1	5.9
Stress, depression, anxiety	7.5	6.8	28.8
Serious mental health problems	0.5	0.6	3.4
Minor illnesses	34.6	23.4	19.5
Respiratory conditions	3.1	4.4	3.1
Gastrointestinal problems	6.8	9.0	6.2
Headaches and migraines	3.8	2.8	3.4
Genito-urinary problems	2.8	2.3	2.6
Heart, blood pressure, circulation problems	1.6	3.5	1.7
Eye, ear, nose and mouth/dental problems	4.0	3.3	2.5
Other (including diabetes)	12.1	14.9	10.9
Prefers not to give details	5.1	4.3	3.9
Unweighted* N*	26,280	8680	2804

Weighted by LFS person-level weights. January 2009–December 2018

Rather than treating people with disabilities and health conditions as a homogenous group, our research adds to this literature by showing how different health problems affect sickness absence to different extents. We use the UK Labour Force Survey (LFS), which is representative of the national population, comprising nearly 1 million observations. It includes information on 17 different health conditions and allows for a measure of sickness absence consistent with official UK statistics, as well as multiple observations per person allowing for longitudinal analysis. This sets our analysis apart from previous studies based largely on cross-sectional data and samples that are not representative of the full working age population.

While long-term health is an important determinant of absenteeism, it is a complex phenomenon and many other factors need to be understood by researchers [9]. Absenteeism is an interesting subject to study for social scientists because an individual's true health state is, to some extent, hidden and this can lead to different behavioural responses. Early theoretical literature [10, 11] shows how employees may have an incentive to ‘shirk’ (take sickness absence when they are not really unwell). Later models incorporate the reverse issue of ‘presenteeism’, the act of coming into work while sick [12–15]. Presenteeism can be a negative outcome for both employers and employees, as attending work while sick may significantly reduce productivity, lengthen recovery rates and, depending on the nature of the illness, put other workers at risk of infection (an issue that as noted has received increased attention during the current pandemic).

Turning to the empirical evidence, it is found that women are at greater risk of absence than men; this is partly, but not fully, explained by occupational segregation [16, 17], and may also be influenced by biological factors [18], and relative preferences for absence as a means of investing in health [19]. The incidence of job strain and informal caregiving responsibilities affect sickness absence disproportionately for women [20], and sickness absence is also higher among parents with young children [21, 22]. At a workplace level, ‘high involvement management’ practices are effective at reducing short-term absence [23], while adverse working conditions and job dissatisfaction predict increased sickness absence [24]. Stress experienced at work [25, 26], time spent commuting [27, 28] and unpleasant or hazardous working conditions [22, 29] are also predictive of higher sickness absence, but job resources (including job control, task significance, supportive behaviour from supervisors and appreciation at work) can help to reduce absenteeism [26].

As absence is, to some extent, a choice, rather than an inevitable consequence of ill-health, many studies show that absence rates are lower where workers have more to lose from being absent. Absence is higher for those with more generous sick pay entitlement [30–32], better perceived job security [33], longer tenure [32, 34], permanent employment contracts [35] and who are members of a trade union [36–38]. Being self-employed is associated with lower absence than being employed [32, 39], while the strictness of the clinician tasked with certifying sickness absence also leads to lower absence [31, 40]. Absence is also significantly higher in the public than the private sector [41]. It is also shown from Spanish data that, while health predicts absenteeism for everybody, the population is heterogeneous with respect to engaging in opportunistic behaviour [42].

## Methods

### Data

The quarterly LFS is the main source of labour market statistics used by the ONS [43, 44]. To provide a representative sample of the UK population, households are sampled from all private addresses in the UK, and all adults within selected households are selected for interview. The LFS uses a rotational sampling design where each household, once selected for interview, is retained in the sample for five consecutive quarters. New households enter the sample every quarter to replace those leaving. As such, the data has a panel structure which we exploit in our analysis. Our sample incorporates all quarters from 2009:Q1 to 2018:Q4 inclusive. To focus on the prime age workforce, and minimise issues around early retirement, we retain observations where the respondent is aged between 21 and 55. The panel is unbalanced due to some individuals not appearing in the survey in every wave or being dropped from our sample in some waves (e.g. if they were not working); although our results are unchanged when we restrict the sample to a balanced panel. The total number of valid observations is 963,144.

### Measures

Following the ONS definition, absenteeism is measured by observing whether actual hours worked in the reference week (the week ending on the Sunday prior to the respondent’s interview) were lower than usual weekly hours and whether this shortfall was due to sickness or injury. This is an established definition of absence used in several other studies based on LFS data, both in the UK and other countries [34, 35, 37, 45]. To capture the salient features of the data (a large number of zero absences and the capping of measured absence at 1 week), we create a binary dependent variable denoting whether or not individual $i$ had at least some sickness absence in the reference week of period $t$ (denoted ${S}_{it}$). Note that this measure captures a snapshot of sickness absence and is not necessarily proportional to absence levels over the whole quarter. It is not clear whether measuring absence in a single week would bias our estimates in either direction. LFS interviews take place throughout the year so the reference week is different for everyone, but it is possible that, rather than taking place at a random time, interviews could be arranged when most convenient for the respondent. This timing could correlate with sickness levels (e.g. delaying the interview if feeling unwell) or working patterns (e.g. being available for interview when not at work due to being on sick leave).

We use a number of different measures to capture the chronic health status of the respondent. First, we construct a dummy variable denoting whether or not the respondent had a chronic health condition, specifically “any physical or mental health conditions or illnesses lasting or expecting to last 12 months or more” (the wording of this question changed slightly in 2013 but we do not expect this to significantly influence our results). Second, we use a set of dummy variables indicating the specific nature of the condition(s), selected from a list of 17 (see Appendix see (Table 7)). Third, we aggregate the conditions into two dummy variables denoting a physical and/or a mental condition respectively (see Appendix see (Table 7)). Fourth, we record the number of different conditions reported by the respondent as a crude measure of the extent of their health problems. Note that, as is usually the case with household survey data, all these measures are based on self-reported health conditions and individuals have not necessarily received a clinical diagnosis of their reported condition.

While reported health conditions are by definition long term in nature (lasting 12 months or more), a large number of individuals in the sample experienced a change in health status across the five consecutive quarters in which they were surveyed. Specifically, 12% of all individuals had some variation in whether they reported a chronic health condition across observations, with 10% experiencing a change in physical health and 3% experiencing a change in mental health. Although these percentages are quite low, the large overall sample gives us statistical power: for example, 7558 individuals experienced a change in mental health. Appendix (see Table 8) shows how these changes vary across the sample. Similar proportions of all demographic groups experience a change in health, although rates are higher for older people and those working in the public sector. This suggests that, although identified from relatively small proportions of the sample, our estimates should be applicable to the population more broadly.

Based on the literature reviewed above, our analysis also controls for a number of other personal and work characteristics expected to affect absenteeism: gender, age, number of dependent children by age, highest educational qualification, marital status, size of workplace, part-time or full-time, whether employment contract is temporary or permanent, length of tenure in current job, industry sector, occupational classification and whether working in the public or private sector. To account for local labour market conditions and macroeconomic factors, we also include the unemployment rate in the respondent’s Local Authority of residence.

### Statistical analysis

We model the effects of health on sickness absence using two different multivariate specifications. Both models are applied to the same sample, which pools all observations over the 10-year period. We first estimate a pooled probit (combining cross-section and time series variation in the dataset) to provide evidence of the extent to which the variation in sickness absence is dependent on health, conditional on other measured characteristics:

1 $$\mathrm{Pr}\left({{S}_{it}=1|H}_{it},{X}_{it}\right) =\Phi ({H}_{it}{\beta }_{1}+{X}_{it}{\gamma }_{1})$$

Here, ${H}_{it}$ denotes the variable or vector of variables describing the health status of individual $i$ in quarter $t$, ${X}_{it}$ contains all other observable variables assumed to influence sickness absence and $\Phi$ denotes the normal cumulative distribution function. We estimate Eq. (1) using the pooled waves of data to derive the association of health with sickness absence after allowing for ${X}_{it}$. If ${X}_{it}$ captured all the relevant characteristics affecting absenteeism, these associations could be interpreted causally. However, they will be biased as causal effects if there is unobserved heterogeneity which influences ${S}_{it}$ and is also correlated with ${H}_{it}$ and ${X}_{it}$. To deal with this, we define ${\nu }_{i}$ as the unobserved characteristics common to an individual but invariant over time. Following Wooldridge [46], we assume that ${\nu }_{i}$ is linearly related to the group means of the explanatory variables such that:

2 $${\nu }_{i}=\psi +{\overline{H} }_{i}\eta +{\overline{X} }_{i}\xi +{a}_{i}$$

Here, ${\overline{H} }_{i}=\frac{1}{T}{\sum }_{t=1}^{T}{H}_{it}$ and ${\overline{X} }_{i}=\frac{1}{T}{\sum }_{t=1}^{T}{X}_{it}$. The error term ${a}_{i}$ can be assumed to be uncorrelated with the group means and normally distributed. We can now add the unobserved heterogeneity into the equation and specify a correlated random effects (CRE) probit that can be estimated consistently using maximum likelihood:

3 $$\mathrm{Pr}\left({{S}_{it}=1|H}_{it},{X}_{it}\right) =\Phi (\psi +{H}_{it}{\beta }_{2}+{\overline{H} }_{i}\eta +{X}_{it}{\gamma }_{2}+{{\overline{X} }_{i}\xi +a}_{i})$$

Since the means control for the measured differences between individuals, adding them separates longitudinal from cross-sectional variation. Hence, the CRE probit estimates the extent to which changes in an individual’s health over time predict changes in their sickness absence. The CRE probit also controls for time-invariant confounders or ‘non-random selection’, because the included means will pick up any other time-invariant differences between individuals which are correlated with the mean characteristics but are otherwise unmeasured. Assuming that the important differences between individuals do not vary over time, the CRE probit estimates can, therefore, be interpreted as ‘causal’ in the sense they control for all confounding factors. Comparing the pooled and CRE estimates can tell us about the role of selection in explaining the relationship between health and sickness absence, as well as the amount of sickness absence that could be eliminated by improved health.

For both models, we present the average marginal effects (AMEs) for all explanatory variables. This is the average predicted percentage point difference in the probability that an individual has some sickness absence (${S}_{it}=1$) as a result of a one-point difference (a zero to one change in the case of binary variables) in the explanatory variable.

## Results

Table 2 describes the distribution of sickness absence (${S}_{it}$) by different characteristics. On average, about 1.5% of prime age workers had some sickness absence during the reference week. The presence of chronic health conditions is associated with a higher probability of sickness absence (2.6%); comparable to the official ONS data [1]. Sickness absence rates are much higher among people with mental health conditions than for those with physical health conditions. About 4.0% of working people suffering from a mental health problem take some sickness absence in any given week, compared with 2.4% for those with a physical condition. The only health condition with higher rates of absence than mental health is progressive illness (5.8%).

**Table 2:** Descriptive statistics

	% having any sickness absence in reference week (${S}_{it}$)	Unweighted *N*	% of total sample
Total sample	1.49	200,377	100.0
No chronic health problem	1.16	153,070	76.4
Any chronic health problem	2.59	47,307	23.6
Any physical health problem	2.43	38,742	19.3
Any mental health problem	4.01	6675	3.3
Specific conditions			
Problems or disabilities (including arthritis or rheumatism) connected with arms or hands	2.79	4599	2.3
Problems or disabilities (including arthritis or rheumatism) connected with legs or feet	2.57	7004	3.5
Problems or disabilities (including arthritis or rheumatism) connected with back or neck	2.84	8591	4.3
Chest or breathing problems, asthma, bronchitis	2.29	9700	4.8
Heart, blood pressure or blood circulation problems	2.09	8400	4.2
Stomach, liver kidney or digestive problems	3.40	5184	2.6
Depression, bad nerves or anxiety	4.26	5488	2.7
Progressive illness not included elsewhere (e.g. cancer, multiple sclerosis, symptomatic HIV, Parkinson’s disease, muscular dystrophy)	5.82	1486	0.7
Gender			
Female	1.84	101,257	50.5
Male	1.16	99,120	49.5
Age group			
Age 21–24	1.43	17,132	8.5
Age 25–34	1.48	54,095	27.0
Age 35–44	1.46	60,753	30.3
Age 45–55	1.53	68,397	34.1
Highest qualification			
Degree or equivalent	1.37	66,322	33.1
Higher education below degree	1.82	19,966	10.0
GCE, A-level or equivalent	1.48	42,496	21.2
GCSE grades A*-C or equivalent	1.55	42,827	21.4
Other qualifications	1.52	17,259	8.6
No qualification	1.33	11,507	5.7
Marital status			
Married/cohabiting/civil partner	1.45	140,984	70.4
Non-married	1.57	59,393	29.6
Number of employees at workplace			
Less than 25	1.34	64,650	32.3
25–49	1.49	26,409	13.2
50–500	1.51	69,547	34.7
Over 500	1.68	39,771	19.8
Part time/full time			
Part-time workers	1.67	44,412	22.2
Full-time workers	1.44	155,965	77.8
Temporary/permanent			
Temporary workers	1.53	8260	4.1
Permanent workers	1.48	192,117	95.9
Length of time in job			
Less than 3 months	1.33	8936	4.5
Between 3 and 6 months	1.54	8463	4.2
Between 6 and 12 months	1.63	13,111	6.5
Between 1 and 2 years	1.62	21,505	10.7
Between 2 and 5 years	1.42	45,986	22.9
Between 5 and 10 years	1.59	40,986	20.5
Between 10 and 20 years	1.42	41,539	20.7
20 years or more	1.36	19,851	9.9
Industry sector			
Manufacturing	1.33	22,385	11.2
Construction	1.10	10,637	5.3
Wholesale and retail trade; repair of motor vehicles and motorcycles	1.36	27,166	13.6
Transportation and storage	1.19	9638	4.8
Accommodation and food service activities	1.31	9386	4.7
Information and communication	1.61	8080	4.0
Financial and insurance activities	1.36	9670	4.8
Professional, scientific and technical activities	1.38	12,396	6.2
Administrative and support service activities	1.41	8585	4.3
Public administration and defence; compulsory social security	1.55	15,152	7.6
Education	1.60	21,755	10.9
Human health and social work activities	1.99	29,844	14.9
Occupation			
Managers, directors and senior officials	0.99	23,310	11.6
Professional occupations	1.49	40,218	20.1
Associate professional and technical occupations	1.50	30,295	15.1
Administrative and secretarial occupations	1.54	24,278	12.1
Skilled trades occupations	1.18	15,635	7.8
Caring, leisure and other service occupations	1.93	18,591	9.3
Sales and customer service occupations	1.67	15,501	7.7
Process, plant and machine operatives	1.50	12,326	6.2
Elementary occupations	1.64	20,223	10.1
Public/private sector			
Public sector	1.84	54,376	27.1
Private sector	1.36	146,001	72.9

Weighted by LFS person-level weights. January 2009–December 2018

Table 2 also shows that absence rates differ across individual and work characteristics. Women and those with ‘other’ higher education have higher absenteeism. Absenteeism is higher in large workplaces, and, in terms of industry, is highest in health and social work and lowest in construction. It is also much higher in the public than the private sector. In terms of occupation, managers, directors and senior officials are least likely to take sickness absence, while absence rates are highest among caring, leisure and other service occupations. Part-time workers have higher absence than those working full-time, while those who have been in their job for less than 3 months have lower absence.

While the figures above suggest that chronic health conditions have a strong effect on absence, this may also reflect other related characteristics. Therefore, it could under- or over-state the relationship among people with comparable characteristics, and similarly it may not reflect the direct causal increase in absence due to a new chronic health condition. To disentangle health from these other factors, we turn to the multivariate models.

The first column of Table 3 shows the results from the pooled probit model. These show that having a chronic health problem is associated with having significantly higher sickness absence controlling for other characteristics. On average, a person with a chronic health condition has a 1.4 percentage point higher probability of taking some absence during a given week than a person with no health problems. Bearing in mind that the overall probability of absenteeism in our sample is 1.5%, this is a large effect. It is also the same as the raw gap in absence rates shown in Table 2, suggesting that none of the difference in absenteeism between those with and without a chronic health condition is explained by other measured characteristics.

**Table 3:** The effect of a chronic health problem on sickness absence

	Average marginal effects	
	Pooled probit	CRE probit
Any health problem	0.0138**	0.0031**
	(0.0004)	(0.0008)
Female	0.0061**	0.0065
	(0.0004)	(0.0036)
Age 21–24	0.0020**	− 0.0002
	(0.0007)	(0.0030)
Age 25–34	0.0028**	− 0.0005
	(0.0005)	(0.0022)
Age 35–44	0.0019**	0.0012
	(0.0004)	(0.0016)
No. of children 0–2	0.0019**	− 0.0036*
	(0.0006)	(0.0014)
No. of children 2–4	0.0004	− 0.0032*
	(0.0004)	(0.0014)
No. of children 5–9	− 0.0010**	− 0.0026*
	(0.0003)	(0.0013)
No. of children 10–15	− 0.0015**	− 0.0020*
	(0.0003)	(0.0011)
Degree or equiv	0.0002	0.0056
	(0.0009)	(0.0031)
Other HE	0.0020*	0.0105**
	(0.0010)	(0.0037)
A-level or equiv	0.0023**	0.0074*
	(0.0009)	(0.0029)
GCSE or equiv	0.0019*	0.0047
	(0.0009)	(0.0025)
Other qualification	0.0025**	0.0050*
	(0.0010)	(0.0025)
Married	− 0.0013**	− 0.0000
	(0.0004)	(0.0022)
< 25 employees	− 0.0026**	− 0.0027
	(0.0005)	(0.0014)
25–49 employees	− 0.0006	− 0.0015
	(0.0006)	(0.0015)
50–499 employees	− 0.0007	− 0.0012
	(0.0004)	(0.0012)
Part time	− 0.0016**	− 0.0003
	(0.0004)	(0.0012)
Temporary	− 0.0002	0.0007
	(0.0007)	(0.0016)
Tenure 3–6 months	0.0031**	0.0052**
	(0.0010)	(0.0013)
Tenure 6–12 months	0.0049**	0.0087**
	(0.0009)	(0.0016)
Tenure 1–2 years	0.0053**	0.0112**
	(0.0009)	(0.0018)
Tenure 2–5 years	0.0044**	0.0102**
	(0.0009)	(0.0018)
Tenure 5–10 years	0.0050**	0.0119**
	(0.0009)	(0.0022)
Tenure 10–20 years	0.0036**	0.0156**
	(0.0009)	(0.0028)
Tenure > 20 years	0.0021*	0.0144**
	(0.0010)	(0.0044)
Mining and quarrying	0.0019	− 0.0117*
	(0.0032)	(0.0047)
Manufacturing	0.0014	− 0.0117*
	(0.0022)	(0.0050)
Electricity and gas	0.0038	0.0017
	(0.0028)	(0.0124)
Water and sewerage	− 0.0000	− 0.0145**
	(0.0028)	(0.0023)
Construction	− 0.0004	− 0.0114**
	(0.0023)	(0.0043)
Wholesale and retail	− 0.0010	− 0.0130*
	(0.0022)	(0.0051)
Transport and storage	− 0.0002	− 0.0118**
	(0.0023)	(0.0042)
Accommodation and food	− 0.0024	− 0.0113**
	(0.0023)	(0.0043)
Information and comm	0.0039	− 0.0109*
	(0.0023)	(0.0045)
Financial and insurance	0.0024	− 0.0098
	(0.0023)	(0.0051)
Real estate	0.0042	− 0.0080
	(0.0026)	(0.0061)
Professional and scientific	0.0023	− 0.0088
	(0.0022)	(0.0053)
Administrative	0.0023	− 0.0105*
	(0.0023)	(0.0045)
Public admin and defence	0.0016	− 0.0116*
	(0.0022)	(0.0049)
Education	0.0026	− 0.0103
	(0.0022)	(0.0059)
Health and social work	0.0017	− 0.0143*
	(0.0022)	(0.0059)
Arts and entertainment	− 0.0011	− 0.0136**
	(0.0025)	(0.0029)
Other services	− 0.0000	− 0.0113**
	(0.0024)	(0.0040)
Households	− 0.0188**	0.0009
	(0.0068)	(0.0241)
Extraterritorial	0.0005	0.0111
	(0.0046)	(0.0218)
Professional	0.0017**	0.0011
	(0.0006)	(0.0017)
Associate professional	0.0028**	0.0023
	(0.0006)	(0.0017)
Administrative	0.0026**	0.0023
	(0.0007)	(0.0020)
Skilled trades	0.0025**	0.0064*
	(0.0008)	(0.0032)
Caring and leisure	0.0037**	0.0024
	(0.0008)	(0.0026)
Sales and customer serv	0.0040**	0.0006
	(0.0008)	(0.0023)
Process, plant, machine	0.0032**	0.0015
	(0.0009)	(0.0028)
Elementary	0.0043**	0.0040
	(0.0008)	(0.0027)
Public sector	0.0026**	0.0030
	(0.0005)	(0.0018)
Local unemp rate	− 0.0001	0.0000
	(0.0001)	(0.0002)
*N*	963,144	963,144

Standard errors (clustered at the individual level) in brackets. CRE probit regressions also include group means of all covariates (not shown). January 2009–December 2018

*p < 0.05

**p < 0.01

The first column of Table 3 also shows that absence is higher for women, those with young children, single people and those outside the older age bracket. The probability of absence is lower in small workplaces and higher for those who have been in their job for more than 3 months; perhaps reflecting the fact that sick leave entitlement is often less generous for new employees. There is some variation across industries and occupations, with absenteeism higher in the public relative to the private sector. This may reflect average health levels in different sectors or different industry norms regarding sickness absence.

Table 4 shows the estimated AMEs of different health conditions on probability of sickness absence. As shown in the first column (for the pooled probit model), both mental and physical health conditions significantly predict absenteeism. However, having a mental health problem is a stronger predictor, with the AME being almost double (1.9 versus 1.1 percentage points). The number of conditions also significantly predicts absence, with each additional condition associated with a 0.5 percentage point increase in absence.

**Table 4:** The effect of different chronic health problems on sickness absence

	Average marginal effects	
	Pooled probit	CRE probit
Any health problem	0.0138**	0.0031**
	(0.0004)	(0.0008)
Number of health problems	0.0050**	0.0014**
	(0.0001)	(0.0003)
Any physical health problem	0.0106**	0.0018*
	(0.0005)	(0.0009)
Any mental health problem	0.0192**	0.0062**
	(0.0012)	(0.0018)
Problems with arms or hands	0.0002	0.0003
	(0.0010)	(0.0019)
Problems with legs or feet	0.0046**	− 0.0001
	(0.0010)	(0.0016)
Problems with back or neck	0.0071**	0.0000
	(0.0010)	(0.0015)
Difficulty in seeing	0.0071**	0.0062
	(0.0021)	(0.0039)
Difficulty in hearing	− 0.0012	0.0072
	(0.0013)	(0.0039)
Speech impediment	0.0038	0.0042
	(0.0047)	(0.0098)
Skin conditions, etc	− 0.0021*	− 0.0027
	(0.0009)	(0.0015)
Chest or breathing problems	0.0038**	0.0021
	(0.0007)	(0.0017)
Heart or blood problems	0.0022**	− 0.0021
	(0.0008)	(0.0014)
Stomach, liver, kidney, etc	0.0103**	0.0034
	(0.0012)	(0.0018)
Diabetes	0.0058**	− 0.0004
	(0.0013)	(0.0037)
Depression, bad nerves, anxiety	0.0176**	0.0059**
	(0.0014)	(0.0019)
Epilepsy	0.0118**	0.0079
	(0.0030)	(0.0087)
Learning difficulties	0.0008	0.0069
	(0.0021)	(0.0053)
Mental illness	0.0035*	0.0003
	(0.0016)	(0.0024)
Progressive illness	0.0242**	0.0098*
	(0.0027)	(0.0041)
Other health problems	0.0089**	0.0031*
	(0.0009)	(0.0014)
*N*	963,144	963,144

Standard errors (clustered at the individual level) in brackets. Four separate regressions shown: any health problem; number of health problems; physical/mental health problems; specific health conditions. *N* is the same for all four regressions. All four regressions include all covariates listed in Table 3 (not shown). CRE probit regressions also include group means of all covariates (not shown). January 2009–December 2018

*p < 0.05

**p < 0.01

Similar to the descriptive statistics, the specific conditions most associated with absenteeism are progressive illnesses and common mental disorders. Most of the other conditions also significantly predict increased absenteeism but a few have no effect (e.g. problems and disabilities connected with arms and hands) and the category bringing together severe disfigurement, skin conditions and allergies is found to have a significant negative effect.

Taking advantage of the longitudinal nature of our data and using the CRE model, the second column of Tables 3 and 4 shows that, once unmeasured differences between individuals are taken into account, the extent to which each of the covariates predicts absenteeism is in general much reduced. Transitioning between having no health problems and having at least one chronic problem is associated with a 0.3 percentage point change in the probability of having some absence, compared with 1.4 percentage points when not controlling for unmeasured differences. Every additional health condition leads to an increase in the probability of absence by 0.1 percentage points. The difference between the pooled and CRE specifications suggests that most of the observed association between chronic health problems and sickness absence is due to other unmeasured individual characteristics, namely confounding factors which raise both the likelihood of absence and of suffering from a chronic health condition. There are many possible explanations but, for example, people with chronic health problems may experience higher levels of deprivation (not directly caused by their condition) or have poorer quality jobs or relationships at work, thus explaining why they may be at more risk of sickness absence regardless of their long-term health. Further, the CRE model isolates recent changes in chronic health conditions (onset or recovery), and the people experiencing these may be systematically different from those with more stable health conditions; for example, the former group may have a lower severity of the condition.

Despite the reduction in the size of the effects in the CRE specification, having a mental health condition continues to be much more predictive of sickness absence than physical health; the AME is more than three times higher (0.6 percentage points versus 0.2). However, there is a high degree of heterogeneity depending on the nature of the physical condition. The single condition that is most predictive of sickness absence in the CRE specification is progressive illness (an AME of 1.0 percentage points). No other individual physical health conditions have a statistically significant effect on probability of absence.

### Robustness checks

We conduct several robustness checks to supplement our main analysis. Unless otherwise stated, the results of these robustness checks are not tabulated but can be provided by the authors on request.

First, we adjust our model to take into account the dynamics of health. We might expect absenteeism rates to respond differently to the onset of and recovery from chronic health conditions. In addition, there may be a delay between acquiring a chronic health condition and an impact on absenteeism. Table 5 shows the results of a model where onset of a health problem is specified separately to recovery from a health problem (to separate onset and recovery, we transform all variables into their changes and estimate the model using OLS). The results show that changes in both directions affect absenteeism. The effects are slightly stronger for an onset of a health condition, but the differences between onset and recovery are not statistically significant, as shown by the *F* test for the equality of coefficients. In Table 6, we present the CRE probit results including a one-quarter lag in health, to assess the additional effects of past health on current absenteeism. In this specification, having a physical health condition in the previous quarter has no additional effect on absenteeism in the current quarter, suggesting that it is current health not past health that is the primary determinant of sickness absence. In contrast, having a mental health condition in the previous quarter has a negative effect on current absenteeism. This could be interpreted as an adaptation effect, whereby a newly acquired mental health condition has an immediate effect on sickness absence but this effect is significantly reduced over time as the condition persists.

**Table 5:** Linear probability model distinguishing onset and recovery

	LPM	Test of coefficient equality (F-statistic)
Onset of any health problem (positive change)	0.0041**	
	(0.0010)	
Recovery from any health problem (negative change)	0.0028**	2.13
	(0.0010)	
Onset of any physical health problem (positive change)	0.0025*	
	(0.0011)	
Recovery from any physical health problem (negative change)	0.0013	1.69
	(0.0011)	
Onset of any mental health problem (positive change)	0.0125**	
	(0.0027)	
Recovery from any mental health problem (negative change)	0.0096**	1.84
	(0.0024)	
*N*	963,144	

Clustered standard errors in bracket. Two separate regressions shown: any health problem; physical/mental health problems. *N* is the same for both regressions. Both regressions include all covariates listed in Table 3 (not shown). January 2009–December 2018. The F-statistic shows the result of a test of equality between the onset and recovery coefficients: no stars denotes that we cannot reject the hypothesis that the two coefficients are equal

*p < 0.05

**p < 0.01

**Table 6:** CRE model with lagged effects

	Average marginal effect
	CRE probit
Any health problem (t)	0.0019
	(0.0010)
Any health problem (t-1)	− 0.0007
	(0.0008)
Any physical health problem (t)	0.0010
	(0.0011)
Any physical health problem (t-1)	0.0003
	(0.0009)
Any mental health problem (t)	0.0059**
	(0.0023)
Any mental health problem (t-1)	− 0.0043**
	(0.0012)
*N*	660,855

Clustered standard errors in brackets. Two separate regressions shown: any health problem; physical/mental health problems. Both regressions include all covariates listed in Table 3 (not shown) and group means of all covariates (not shown). January 2009–December 2018

*p < 0.05

**p < 0.01

Second, we alter the definition of absenteeism to include only those who had been off work for the entire reference week due to sickness absence. This tells a similar story in terms of the difference between physical and mental health. On average, a change in physical health does not result in a significant change in this longer term absence while the effect of a change in mental health remains positive and significant. We also specify absenteeism as a continuous variable, defined as the number of hours in the reference week that the individual has been absent due to sickness or injury. Due to absence hours in a given week being a highly censored variable (most people have zero hours), we use a tobit model with a lower bound censor of 0 and find that this specification yields similar results to our main model. Health problems affect the underlying number of hours of sickness absence, as well as whether a person has any absence, with mental health problems again having a larger effect than physical health problems.

Third, we change the definition of the health variable to include only those with more severe conditions. In the LFS, respondents with a health condition are asked whether this limits the amount or kind of work that they can do. If we restrict our health variable only to those who have a work-limiting health condition, the results are qualitatively similar but no longer significant in the CRE probit model. This is likely due to smaller cell sizes as a large proportion of people with work-limiting health conditions are not in employment, and therefore, not in our sample.

Fourth, to explore heterogeneity in the relationship by gender we conduct separate regressions for males and females. We find that health problems affect absenteeism to a similar extent for both sexes and there are no significant differences between them.

Finally, to check whether non-random attrition (e.g. job loss related to absenteeism) might affect the estimates, we repeat the main specifications on a balanced panel only (by removing individuals that are not observed in all five waves). This corroborates our main findings.

## Discussion

The findings reported here suggest that, somewhat unsurprisingly, people with a chronic health condition are more likely to experience sickness absence, an outcome that is costly to employers and the economy and can be disadvantageous to individuals. The more important result is that a change in mental health has a much larger effect on absenteeism than a change in physical health. In percentage point terms, the mental health effect is three times as large (0.6 percentage points versus 0.2 percentage points). Taking existing absenteeism rates as the baseline, the gap is somewhat smaller in proportionate terms. Thus, our results suggest that recovering from a mental health condition would reduce absence rates by 15% (from 4.0 to 3.4% on average) while recovering from a physical health condition would reduce absence rates by 8% (from 2.4 to 2.2% on average). Nonetheless, this still suggests that the mental health effect is nearly double the physical health effect. These results confirm previous findings from Australia [4, 5] that a change in mental health is a significant predictor of workplace absenteeism, but for the first time we show the size of this effect relative to a change in physical health. This result for sickness absence is consistent with the health-related causes of permanent labour market exit in the UK, where mental health problems are now the most prevalent reason for claiming Employment Support Allowance [47].

There are a few limitations to our analysis which serve to caveat our results. First, the data allow us to identify discrete transitions in long-term health but do not provide a measurement of the severity of health conditions. We implicitly assume that a transition into poor mental health is of equal severity to a transition into poor physical health, while in reality this may not be the case. Second, our results are based on a selected sample who remain in employment. For some individuals, a deterioration in health may lead to an exit from employment (similarly, an improvement in health may result in entry). In a sense, exit from employment is a more extreme form of absenteeism so by excluding such individuals from our analysis, we may be underestimating the full labour market effects of health changes.

The implications from this research are twofold. First, our findings provide clear evidence that investment in health will deliver significant benefits to the UK economy due to reduced absenteeism. However, not all investments should be expected to have an equal return. For example, people experiencing an onset of depression or anxiety are estimated to be 0.6 percentage points more likely to have some absence in any given week. We also know from the data that a person with this type of common mental disorder who has some absence takes on average 3 days off during the week. This implies that alleviating these mental health problems could save about 1 day of absence per worker per year (assuming 50 working weeks in a year); to the extent that sickness absence is a precursor to permanent labour market exit, this will also have benefits in reducing premature exit due to mental ill-health. The prevention and treatment of common physical conditions would, on the other hand, deliver much more modest returns.

Second, while healthcare interventions have an important role to play, disability and chronic health conditions will inevitably persist in the working age population. Following the COVID-19 pandemic, the UK may experience worsening health, especially mental health, long after the immediate crisis has passed [48]. A key challenge for policy is to ensure that health is not a barrier to accessing suitable and sustainable work. This challenge is particularly acute for people with poor mental health, as this subset of the working age disabled population has among the lowest rates of employment in the UK. Our findings highlight the fact that, everything else being equal, increasing the employment rates of people with chronic mental health conditions is likely to lead to increased rates of sickness absence overall, thus negating some of the economic gains of higher employment. Policies aimed at improving the employability of people with poor mental health must include measures to ensure that workplaces are healthy environments that can adapt to the needs of those most at risk of persistent absence.

## Data Availability

Not applicable.

## Appendix

See Table 7 and 8

**Table 7:** List of health conditions included in the LFS

Description of condition	Mental or physical [49]
Problems or disabilities (including arthritis or rheumatism) connected with arms or hands	Physical
Problems or disabilities (including arthritis or rheumatism) connected with legs or feet	Physical
Problems or disabilities (including arthritis or rheumatism) connected with back or neck	Physical
Difficulty in seeing (while wearing spectacles and contact lenses)	Physical
Difficulty in hearing	Physical
A speech impediment	Physical
Severe disfigurement, skin conditions, allergies	Physical
Chest or breathing problems, asthma, bronchitis	Physical
Heart, blood pressure or blood circulation problems	Physical
Stomach, liver kidney or digestive problems	Physical
Diabetes	Physical
Depression, bad nerves or anxiety	Mental
Epilepsy	Physical
Severe or specific learning difficulties (mental handicap)	Mental
Mental illness, or suffer from phobia, panics or other nervous disorders	Mental
Progressive illness not included elsewhere (e.g. cancer, multiple sclerosis, symptomatic HIV, Parkinson’s disease, and muscular dystrophy)	Physical
Other health problems or disabilities	Neither

**Table 8:** Number of people in study sample reporting a variation in health across survey quarters

	Change in any health problem		No change in any health problem		Change in any physical health problem		No change in any physical health problem		Change in any mental health problem		No change in any mental health problem	
	Number	%	Number	%	Number	%	Number	%	Number	%	Number	%
Total sample	32,428	11.7	245,018	88.3	28,382	10.2	249,064	89.8	7558	2.7	269,888	97.3%
Gender:												
Female	16,997	12.2	122,481	87.8	14,396	10.3	125,082	89.7	4658	3.3	134,820	96.7%
Male	15,431	11.2	122,537	88.8	13,986	10.1	123,982	89.9	2900	2.1	135,068	97.9%
Age group												
Age 21–24	2037	7.3	25,763	92.7	1609	5.8	26,191	94.2	669	2.4	27,131	97.6%
Age 25–34	6764	8.6	71,741	91.4	5632	7.2	72,873	92.8	1840	2.3	76,665	97.7%
Age 35–44	9818	12.0	72,170	88.0	8492	10.4	73,496	89.6	2274	2.8	79,714	97.2%
Age 45–55	13,809	15.5	75,344	84.5	12,649	14.2	76,504	85.8	2775	3.1	86,378	96.9%
Highest qualification												
Degree or equivalent	10,083	11.0	81,900	89.0	8635	9.4	83,348	90.6	2165	2.4	89,818	97.6%
Higher education below degree	3548	13.0	23,678	87.0	3136	11.5	24,090	88.5	761	2.8	26,465	97.2%
GCE, A-level or equivalent	7059	11.9	52,200	88.1	6171	10.4	53,088	89.6	1723	2.9	57,536	97.1%
GCSE grades A*-C or equivalent	7220	12.3	51,383	87.7	6390	10.9	52,213	89.1	1818	3.1	56,785	96.9%
Other qualifications	2731	11.1	21,900	88.9	2453	10.0	22,178	90.0	612	2.5	24,019	97.5%
No qualification	1787	11.4	13,957	88.6	1597	10.1	14,147	89.9	479	3.0	15,265	97.0%
Marital status												
Married/cohabiting/civil partner	23,176	12.2	166,230	87.8	20,468	10.8	168,938	89.2	4588	2.4	184,818	97.6%
Non-married	9252	10.5	78,788	89.5	7914	9.0	80,126	91.0	2970	3.4	85,070	96.6%
Number of employees at workplace												
Less than 25	10,318	11.5	79,765	88.5	8993	10.0	81,090	90.0	2664	3.0	87,419	97.0%
25–49	4390	12.0	32,142	88.0	3820	10.5	32,712	89.5	1083	3.0	35,449	97.0%
50–500	11,195	11.7	84,819	88.3	9858	10.3	86,156	89.7	2495	2.6	93,519	97.4%
Over 500	6525	11.9	48,292	88.1	5711	10.4	49,106	89.6	1316	2.4	53,501	97.6%
Part time/full time												
Part-time workers	7410	12.4	52,273	87.6	6285	10.5	53,398	89.5	2379	4.0	57,304	96.0%
Full-time workers	25,018	11.5	192,745	88.5	22,097	10.1	195,666	89.9	5179	2.4	212,584	97.6%
Temporary/permanent												
Temporary workers	1231	10.0	11,058	90.0	1044	8.5	11,245	91.5	405	3.3	11,884	96.7%
Permanent workers	31,197	11.8	233,960	88.2	27,338	10.3	237,819	89.7	7153	2.7	258,004	97.3%
Length of time in job												
Less than 3 months	1385	10.4	11,921	89.6	1159	8.7	12,147	91.3	408	3.1	12,898	96.9%
Between 3 and 6 months	1277	10.1	11,398	89.9	1068	8.4	11,607	91.6	390	3.1	12,285	96.9%
Between 6 and 12 months	2012	10.4	17,363	89.6	1725	8.9	17,650	91.1	565	2.9	18,810	97.1%
Between 1 and 2 years	3227	10.4	27,943	89.6	2794	9.0	28,376	91.0	871	2.8	30,299	97.2%
Between 2 and 5 years	7000	10.8	57,975	89.2	6042	9.3	58,933	90.7	1706	2.6	63,269	97.4%
Between 5 and 10 years	6481	11.6	49,194	88.4	5707	10.3	49,968	89.7	1456	2.6	54,219	97.4%
Between 10 and 20 years	7149	13.1	47,533	86.9	6361	11.6	48,321	88.4	1476	2.7	53,206	97.3%
20 years or more	3897	15.2	21,691	84.8	3526	13.8	22,062	86.2	686	2.7	24,902	97.3%
Industry sector												
Manufacturing	3606	11.8	26,934	88.2	3242	10.6	27,298	89.4	619	2.0	29,921	98.0%
Construction	1622	10.9	13,304	89.1	1459	9.8	13,467	90.2	256	1.7	14,670	98.3%
Wholesale and retail trade; repair of motor vehicles	4154	10.9	33,833	89.1	3659	9.6	34,328	90.4	1111	2.9	36,876	97.1%
Transportation and storage	1572	11.9	11,590	88.1	1439	10.9	11,723	89.1	292	2.2	12,870	97.8%
Accommodation and food service activities	1320	9.6	12,492	90.4	1114	8.1	12,698	91.9	395	2.9	13,417	97.1%
Information and communication	1290	11.5	9890	88.5	1123	10.0	10,057	90.0	240	2.1	10,940	97.9%
Financial and insurance activities	1426	10.5	12,122	89.5	1259	9.3	12,289	90.7	277	2.0	13,271	98.0%
Professional, scientific and technical activities	1897	10.9	15,550	89.1	1684	9.7	15,763	90.3	385	2.2	17,062	97.8%
Administrative and support service activities	1309	10.8	10,806	89.2	1115	9.2	11,000	90.8	365	3.0	11,750	97.0%
Public admin and defence; compulsory social security	2597	12.6	18,018	87.4	2309	11.2	18,306	88.8	577	2.8	20,038	97.2%
Education	3838	13.2	25,221	86.8	3304	11.4	25,755	88.6	959	3.3	28,100	96.7%
Human health and social work activities	5319	13.0	35,636	87.0	4564	11.1	36,391	88.9	1455	3.6	39,500	96.4%
Occupation												
Managers, directors and senior officials	3833	11.7	28,961	88.3	3436	10.5	29,358	89.5	598	1.8	32,196	98.2%
Professional occupations	6494	11.9	47,939	88.1	5637	10.4	48,796	89.6	1357	2.5	53,076	97.5%
Associate professional and technical occupations	4851	11.5	37,354	88.5	4185	9.9	38,020	90.1	1054	2.5	41,151	97.5%
Administrative and secretarial occupations	3974	12.0	29,127	88.0	3411	10.3	29,690	89.7	1017	3.1	32,084	96.9%
Skilled trades occupations	2451	11.2	19,360	88.8	2229	10.2	19,582	89.8	457	2.1	21,354	97.9%
Caring, leisure and other service occupations	3274	12.7	22,413	87.3	2827	11.0	22,860	89.0	990	3.9	24,697	96.1%
Sales and customer service occupations	2436	11.2	19,355	88.8	2093	9.6	19,698	90.4	830	3.8	20,961	96.2%
Process, plant and machine operatives	1963	11.6	14,972	88.4	1807	10.7	15,128	89.3	370	2.2	16,565	97.8%
Elementary occupations	3152	11.0	25,537	89.0	2757	9.6	25,932	90.4	885	3.1	27,804	96.9%
Public/private sector												
Public sector	9654	13.1	63,894	86.9	8375	11.4	65,173	88.6	2297	3.1	71,251	96.9%
Private sector	22,774	11.2	181,124	88.8	20,007	9.8	183,891	90.2	5261	2.6	198,637	97.4%

Refers to the characteristics of individuals in the first wave in which they are observed. Some industry sectors not shown due to small sample sizes
